# Nuclear Microsatellite Primers for the Endangered Relict Fir, *Abies pinsapo* (Pinaceae) and Cross-Amplification in Related Mediterranean Species

**DOI:** 10.3390/ijms131114243

**Published:** 2012-11-05

**Authors:** Jose M. Sánchez-Robles, Francisco Balao, Juan L. García-Castaño, Anass Terrab, Laura Navarro-Sampedro, Salvador Talavera

**Affiliations:** 1 Departamento de Biología Vegetal y Ecología, Universidad de Sevilla, Apdo. 1095, Sevilla E-41080, Spain; E-Mails: fbalao@us.es (F.B.); jlgc@us.es (J.L.G.-C.); anass@us.es (A.T.); stalavera@us.es (S.T.); 2 Centro de Investigación, Tecnología e Innovación (CITIUS), Universidad de Sevilla, Apdo. 1095, Sevilla E-41080, Spain; E-Mail: lauranavarro@us.es

**Keywords:** *Abies*, genetic conservation, mediterranean basin, nSSR, Pinaceae, population genetics

## Abstract

Twelve nuclear microsatellite primers (nSSR) were developed for the endangered species *Abies pinsapo* Boiss. to enable the study of gene flow and genetic structure in the remaining distribution areas. Microsatellite primers were developed using next-generation sequencing (454) data from a single *Abies pinsapo* individual. Primers were applied to thirty individuals from the three extant localities. The number of alleles per locus ranged from one to four. Cross-amplification was tested for other *Abies* species from the Mediterranean Basin, and most of the loci showed higher polymorphisms in the Mediterranean species than in *A. pinsapo*. These microsatellite markers provide tools for conservation genetic studies in *Abies pinsapo* as well other *Abies* species from the Mediterranean Basin.

## 1. Introduction

*Abies pinsapo* Boiss. is a tertiary relict fir species, endemic to three localities in southern Spain: Sierra de Grazalema Natural Park, Sierra de las Nieves Natural Park and the Sierra Bermeja Natural Area. Listed as an endangered species by the International Union for Conservation of Nature (IUCN), *A. pinsapo* forms woodlands that have a special ecological importance for many organisms [1]. Their current limited range makes them highly vulnerable to disturbance [2].

There are a few genetic studies focused on *Abies pinsapo*[3–6] but none used nuclear DNA molecular markers. Nuclear microsatellites (nSSR) are highly polymorphic markers, codominant and usually selectively neutral, making them suitable for the analysis of small-scale genetic diversity [7]. Previous authors have developed primers for nSSR in others *Abies* species, amplifying some of them in *Abies pinsapo*[8,9]. In this study, we report the isolation and characterization of 12 nuclear microsatellites by the efficient and quick 454 sequencing technique. Primers developed for these markers were applied in individuals from the three main mountain areas that encompass the whole distribution range. Additionally, these markers were evaluated in the 11 remaining *Abies* species from the Mediterranean Basin.

## 2. Results and Discussion

### 2.1. Primers for *Abies pinsapo*

Twelve high-quality loci were tested in 30 individuals from the three *Abies pinsapo* populations studied (Table 1). Three of them (*Pin14*, *Pin22* and *Pin25*) were monomorphic. The number of alleles in the polymorphic loci ranged from two to five (mean ± SE: 2.074 ± 0.150). Their observed and expected heterozygosities (*H*_o_ and *H*_e_) ranged both from 0.100 to 0.600. Null allele frequency (*N*_a_) ranged from 0.000 to 0.907. After Bonferroni correction for multiple comparisons (adjusted *α* = 0.002), no locus showed a significant deviation from expectations under Hardy-Weinberg equilibrium (HWE) and no linkage disequilibrium (LD) was detected for the 36 paired loci comparisons (Table 2).

### 2.2. Cross-Amplification in Other Mediterranean *Abies* Species

Primer amplifications in other Mediterranean *Abies* species were successful in most cases. Seven primers amplified in all species, *Pin8* did not amplify in any of them and the remaining four amplified at least in three species. For several loci, there was a lower number of alleles in *A. pinsapo* than in the other Mediterranean species, in spite of using fewer individuals for the latter (three individuals in *A. nebrodensis* and five individuals in the rest of them); see Table 3. This low number of alleles in *Abies pinsapo* could be due to a low effective population size [10].

## 3. Experimental Section

### 3.1. DNA Extraction, 454 Sequencing and Microsatellite Discovery

Genomic DNA was extracted from leaf tissue from a single *Abies pinsapo* individual using a QIAGEN DNeasy Plant Mini Kit (QIAGEN, Valencia, CA, USA). A size selected DNA library was built and enriched for microsatellites using Dynabeads, as by Glenn and Schable [11] and sequenced on a 454 Genome Sequencer FLX System (454 Life Sciences, a Roche company, Branford, CT, USA) at the Savannah River Ecology Laboratory (Aiken, SC, USA). The 454 sequencing technique is described in detail in Abdelkrim *et al.*[12] and Lance *et al.*[13]. Development of microsatellites via “next generation sequencing” can be quicker, cheaper and it recovers more loci than enrichment methods. Moreover, this technique is more suitable for this purpose because of the large average size of the fragments obtained, which facilitates primer design [14]. CAP3 [15] was used to assemble sequences at 98% sequence identity using a minimal overlap of 75 bp. Sequence data were screened for di-, tri- and tetra-nucleotide repeats using the program MSATCOMMANDER version 0.8.1 [16] with minimum repeats set to eight, seven and six respectively, and primers were designed with Primer3 [17]. Two default 5′-tails (CAG: 5′-CAGTCGGGCGTCATCA-3′ and M13R: 5′-GGAAA CAGCTATGACCAT-3′) options were considered for designed primers, as with Faircloth [16], to allow a cheaper labeling technique than the direct labeling of the primers [18], as the inclusion of the 5′-tail allows the use of a third primer in the polymerase chain reaction (PCR) (M13R or CAG) that is fluorescently labeled for detection in the DNA Analyzer sequencer [19]. Additionally, a not-tagged primer from a primer pair was designed with a 5′-GTTT tail to promote adenylation and thus facilitate genotyping [20]. Primers could be designed for 497 of the 3617 sequences obtained. Out of these 497 primer pairs, we removed 196 with low quality (*i.e.*, redundant, and with high likelihood of forming secondary structures such as hairpins and self or primer dimers). Therefore 301 primers with high quality were selected.

### 3.2. Primer Amplification and Quality Test

Preliminary screenings from 50 loci (out of the 301 selected) were done to test the amplification quality using two previously obtained high quality DNA samples. Of these, 31 loci amplified well, but 19 primer pairs produced unclear patterns in the electropherograms. Conditions for PCR amplification were an initial denaturation step of 5 min at 94 °C, followed by five cycles of 94 °C for 30 s, 60 °C for 30 s, and 72 °C for 30 s; 21 cycles of 94 °C for 30 s, 60 °C for 30 s (decreased by 0.5 °C per cycle), and 72 °C for 30 s; 15 cycles of 94 °C for 30 s, 50 °C for 30 s and 72 °C for 30 s; and a final step of 8 min at 72 °C. PCR reactions were carried out using approximately 50 ng of genomic DNA in a total volume of 20 μL, containing 2 μL of PCR Buffer 1× (0.2 mM MgCl_2_), 2 μL of dNTP 0.1 mM (0.025 mM each), 0.5 μL of BSA (0.025 mg/mL), 0.2 μL of i-Start Taq DNA polymerase (1 U/μL) (iNtRON Biotecnology Inc., Sungnam, Korea), 1.25 μL of primer with 5′-GTTT tail (0.25 μM), 0.3 μL of primer with 5′-CAG or M13R tail (0.06 μM) and 0.5 μL of universal primer with 6-carboxyfluorescein (FAM), nitrobenzoxadiazolyl (NED), or VIC fluorescent label (0.25 μM). PCR products were run on a 3730 DNA Analyzer sequencer (Applied Biosystem, Foster City, CA, USA) and sized with LIZ 500 standard (Applied Biosystem, Foster City, CA, USA). Fragments were analyzed using GENEMARKER version 1.8 (SoftGenetics, State College, PA, USA).

### 3.3. Genotyping and Cross-Amplification

Twelve high-quality loci were tested in 30 individuals, 10 from each of the three unique *A. pinsapo* localities (Sierra de Grazalema Natural Park, Sierra de las Nieves Natural Park, and Sierra Bermeja Natural Area). Cross-amplification tests for all loci were performed with the 11 remaining *Abies* species from the Mediterranean Basin (see Supplementary Information), using five samples per species across their distribution ranges (three individuals for the micro-endemic *A. nebrodensis*).

### 3.4. Genetic Analyses

Observed heterozygosity (*H*_o_), expected heterozygosity (*H*_e_), null allele frequency (*A*_n_) and Hardy-Weinberg equilibrium test (HWE) were obtained for each locus and population in CERVUS 3.0.3 software [21]. A test for genotypic linkage disequilibrium was conducted with GENEPOP 4.0.10 software [22].

## 4. Conclusions

We present twelve nuclear microsatellite loci for the Spanish fir *Abies pinsapo* Boiss. These loci may be useful for conservation genetic studies and they will be used to improve our understanding of gene movement between the relict populations of this species. Information derived from genetic studies may contribute to the development of more effective plans for the recovery and management of this endangered species. Furthermore, since most of the primers developed for *Abies pinsapo* amplified successfully in the other Mediterranean *Abies* species, they are suitable for both intra- and inter-specific studies.

## Supplementary Information



## Figures and Tables

**Table 1:** Details of 12 novel microsatellite primers developed in *Abies pinsapo*.

Locus	Primer sequences (5′-3′) a	Repeat motif	Size (bp)	*T*_a_ (°C)	*N*_a_	GenBank Accession No.
*Pin8*	F: ‡TGATTTATCCTTCGGTCTTG R: *AAAGGGCTATGTTTGAATTG	(CTT)11	258	50	3	JX473825
*Pin14*	F: ‡CAAATGTTGCAATGTAGGAC R: *CCGAATATCTTGCTAGTTGTC	(AGA)8	249	50	1	JX473826
*Pin17*	F: ‡TCCGATCCAATAGAAGATTC R: *AAAGGTTGAATCAATGATCC	(ATTC)8	428	50	2	JX473827
*Pin20*	F: ‡ATACAGTTATTGGCGACTCC R:*GTGAGGCGTGTGTACAATC	(CATA)8	296	50	4	JX473828
*Pin22*	F: *GAGTCACATGCTTTGGTGAG R: ‡TTACCACTCAAGGCCATTAC	(CAT)7	108	50	1	JX473829
*Pin25*	F: ‡CCCTAATTCAATGCAATTATC R: *GCATCCTGTAGAGCAACTTG	(TATG)8	167	50	1	JX473830
*Pin29*	F: ‡TGATTTATCCTTCGGTCTTG R: *AAAGGGCTATGTTTGAATTG	(CTT)11	257	50	2	JX473831
*Pin32*	F: ‡CAGCCCAAATAATTCTCTTC R: *TAGCCTTTCTTGATGGAGAG	(CAT)7	254	50	2	JX473832
*Pin44*	F: *GAACGATACCATTGCATCTC R: †ACATGCTTTCTATTTCCAATC	(AC)11	138	50	2	JX473833
*Pin48*	F: *CCATGGACTTCGGTAATATC R: ‡TCATGACAACAAGCGAGAAC	(GT)10	189	50	5	JX473834
*Pin49*	F: †AAGCTGGATAATGAAAGGAC R: *GCAAATTGGTCTTTAACTCC	(AG)10	173	50	2	JX473835
*Pin52*	F: ‡AACACCAGGCATTCACATC R: *ACTAGCTAAGCAACCACCTC	(CA)11	274	50	2	JX473836

Note: F = forward primer; R = reverse primer; *T*_a_ = annealing temperature; *N*a = number of alleles;

a * indicates GTTT tag; † indicates CAG tag (5′-CAGTCGGGCGTCATCA-3′), ‡ indicates M13R tag (5′-GGAAACAGCTATGACCAT-3′).

**Table 2:** Results of primer screening in three *Abies pinsapo* populations.

	Sierra de las Nieves (*N* = 10)					Grazalema (*N* = 10)					Sierra Bermeja (*N* = 10)				
Locus	*A*	*H*_o_	*H*_e_	HWE	*A*_n_	*A*	*H*_o_	*H*_e_	HWE	*A*_n_	*A*	*H*_o_	*H*_e_	HWE	*A*_n_
*Pin8*	2	0.400	0.442	0.880	0.024	2	0.100	0.479	0.014	0.640	3	0.500	0.416	0.774	0.000
*Pin14*	1	0.000	0.000	-	-	1	0.000	0.000	-	-	1	0.000	0.000	-	-
*Pin17*	1	0.000	0.000	-	-	1	0.000	0.000	-	-	2	0.100	0.100	0.868	0.000
*Pin20*	3	0.300	0.416	0.667	0.128	2	0.000	0.189	0.002	0.907	1	0.000	0.000	-	-
*Pin22*	1	0.000	0.000	-	-	1	0.000	0.000	-	-	1	0.000	0.000	-	-
*Pin25*	1	0.000	0.000	-	-	1	0.000	0.000	-	-	1	0.000	0.000	-	-
*Pin29*	2	0.400	0.442	0.880	0.024	2	0.100	0.479	0.014	0.640	2	0.500	0.395	0.292	0.000
*Pin32*	1	0.000	0.000	-	-	1	0.000	0.000	-	-	2	0.200	0.442	0.098	0.355
*Pin44*	2	0.600	0.505	0.429	0.000	2	0.400	0.337	0.429	0.000	2	0.400	0.526	0.527	0.111
*Pin48*	4	0.400	0.600	0.640	0.192	3	0.500	0.542	0.179	0.000	4	0.500	0.432	0.981	0.000
*Pin49*	2	0.300	0.479	0.281	0.205	2	0.500	0.521	0.975	0.000	2	0.400	0.337	0.429	0.000
*Pin52*	2	0.100	0.100	0.868	0.000	2	0.200	0.189	0.725	0.000	2	0.200	0.442	0.098	0.355

Note: *N* = simple size; *A* = number of alleles; *H*_o_ = observed heterozygosity; *H*_e_ = expected heterozygosity; *A*_n_ = null allele frequency; HWE = Hardy-Weinberg equilibrium test (*p*-values).

**Table 3:** Screening of primers developed in *Abies pinsapo* in the 11 remaining *Abies* species from the Mediterranean Basin.

Species	*Pin14*		*Pin17*		*Pin20*		*Pin22*		*Pin25*		*Pin29*		*Pin32*		*Pin44*		*Pin48*		*Pin49*		*Pin52*	

	*P*	*A*	*P*	*A*	*P*	*A*	*P*	*A*	*P*	*A*	*P*	*A*	*P*	*A*	*P*	*A*	*P*	*A*	*P*	*A*	*P*	*A*
*A. alba*	(0/5)	-	(5/5)	1	(5/5)	1	(5/5)	5	(5/5)	2	(5/5)	1	(5/5)	2	(5/5)	3	(5/5)	3	(5/5)	3	(5/5)	1
*A. boisii-regis*	(5/5)	1	(5/5)	3	(5/5)	5	(5/5)	2	(5/5)	8	(5/5)	5	(5/5)	5	(5/5)	4	(5/5)	6	(5/5)	2	(5/5)	5
*A. bornmuelleriana*	(0/5)	-	(0/5)	-	(5/5)	2	(1/5)	1	(5/5)	1	(5/5)	2	(1/5)	1	(5/5)	2	(5/5)	6	(5/5)	1	(5/5)	4
*A. cephalonica*	(0/5)	-	(0/5)	-	(5/5)	3	(4/5)	5	(5/5)	7	(5/5)	3	(0/5)	-	(5/5)	5	(5/5)	5	(5/5)	3	(4/5)	4
*A. cilicica*	(0/5)	-	(1/5)	1	(5/5)	1	(0/5)	-	(5/5)	3	(5/5)	2	(0/5)	-	(5/5)	1	(5/5)	7	(5/5)	1	(5/5)	4
*A. equi-trojani*	(0/5)	-	(1/5)	1	(5/5)	3	(5/5)	3	(5/5)	2	(4/5)	3	(0/5)	-	(5/5)	2	(5/5)	4	(5/5)	1	(5/5)	5
*A. marocana*	(4/5)	2	(1/5)	1	(5/5)	3	(3/5)	4	(5/5)	1	(5/5)	3	(5/5)	2	(5/5)	2	(5/5)	4	(5/5)	1	(5/5)	2
*A. nebrodensis*	(0/3)	-	(0/3)	-	(3/3)	1	(1/3)	1	(3/3)	2	(3/3)	2	(3/3)	3	(3/3)	2	(3/3)	1	(3/3)	2	(3/3)	3
*A. nordmanniana*	(0/5)	-	(0/5)	-	(5/5)	2	(0/5)	-	(5/5)	4	(4/5)	3	(0/5)	-	(5/5)	3	(5/5)	6	(5/5)	2	(5/5)	5
*A. numidica*	(4/5)	1	(4/5)	2	(3/5)	3	(0/5)	-	(5/5)	3	(1/5)	1	(4/5)	1	(5/5)	2	(5/5)	4	(5/5)	2	(3/5)	2

Note: *P =* number of individuals that amplified in the sample; *A* = number of alleles.
