# Supplementary material for: Nuclear Microsatellite Primers for the Endangered Relict Fir, Abies pinsapo (Pinaceae) and Cross-Amplification in Related Mediterranean Species
Source: Int J Mol Sci. 2012 Nov 5;13(11):14243–50. doi: 10.3390/ijms131114243 (PMC3509577; doi:10.3390/ijms131114243)
Supplement: Supplementary file 1 [file ijms-13-14243-s001.pdf]

Supplementary Information

Table 1. Geographical data of *Abies* populations used in this study and Herbarium Voucher from University of Seville. *N* = number individuals sampled.

Species	Population	Country	Coordinates	*N*	Voucher
Abies pinsapo	Sierra de las Nieves	Spain	36°41'47"N, 05°01'37"E	10	SEV77675
	Sierra de Grazalema	Spain	36°46'13"N, 05°24'52"E	10	SEV68656
	Sierra Bermeja	Spain	36°29'13"N, 05°12'26"E	10	SEV35720
A. alba	Mata de Valencia	Spain	42°38'01"N, 01°04"45"E	2	SEV264618
	Sierra de Montseny	Spain	41°46'38"N, 02°26'44"E	1	SEV264619
	Rothwald	Austria	47°45'18"N, 15°02'46"E	2	-
A. boisii-regis	Tríkala	Turkey	39°39'11"N, 21°22'21"E	3	SEV278034
	Metsovo	Turkey	39°45'15"N, 21°10'52"E	2	SEV278033
A. bornmuelleriana	Kostamonu	Turkey	41°03'21"N, 33°27'01"E	3	SEV278038
	Sinop	Turkey	41°25'38"N, 34°34'08"E	2	SEV278037
A. cephalonica	Oros Aenos Kefalonia	Greece	38°09'24"N, 20°39'41"E	2	-
	Kalavryta	Greece	38°04'23"N, 22°07'30"E	2	SEV278031
	Oros Pateras	Greece	38°08'38"N, 23°18'40"E	1	SEV256671
A. cilicica	Gidengelmez Dağları Milli Park	Turkey	37°12'06"N, 31°48'06"E	1	SEV273455
	Isparta. Asagigokdere	Turkey	37°35'20"N, 30°49'01"E	2	SEV273454
	Antalya	Turkey	37°11'54"N, 31°08'12"E	2	SEV273452
A. equi-trojani	Kazdagi	Turkey	39°42'00"N, 26°49'48"E	5	SEV278039
A. nebrodensis	Vallone Madonna degli Angeli	Italy	37°52'32"N, 14°02'25"E	3	SEV256715
A. nordmanniana	Región de Giresun	Turkey	40°24'37"N, 38°23'58"E	3	SEV278035
	Ordu	Turkey	40°35'17"N, 37°40'53"E	2	SEV278036
A. numidica	Edough	Algeria	36°33'00"N, 05°28'00"E	5	-
A. tazaotana	JebelTazaot	Morocco	35°20'06"N, 05°09'31"W	5	SEV277703
A. marocana	Talassemtane	Morocco	35°10'43"N, 05°14'03"W	3	SEV165953
	Adeldal	Morocco	35°11'05"N, 05°05'14"W	2	SEV165953

© 2012 by the authors; licensee MDPI, Basel, Switzerland. This article is an open access article distributed under the terms and conditions of the Creative Commons Attribution license (http://creativecommons.org/licenses/by/3.0/).
